# Editorial: In honor of Dr. Bruce Ames: innovations in mutagenesis and DNA repair

**DOI:** 10.3389/fmolb.2026.1824006

**Published:** 2026-03-27

**Authors:** Cecilia Giulivi, Maret G. Traber

**Affiliations:** 1 Department of Molecular Biosciences, School of Veterinary Medicine, University of California, Davis, United States; 2 Linus Pauling Institute, College of Health, Oregon State University, Corvallis, OR, United States

**Keywords:** Ames assay, Ames bacterial mutagenicity test, bacterial reversion assay, aging, duplex sequencing, regulatory science, zinc

## Introduction

Scientific legacies are not made based just on the tools and methods the scientists develop, but more importantly on the conceptual breakthroughs they inspire. Few examples highlight this concept better than Bruce Ames’ work. His creation of the Ames test transformed mutagenicity testing worldwide, creating a practical, scalable link between molecular genetics and public health. However, the intellectual movement he started continues to evolve—now reaching into the age of ultra-sensitive genome-wide sequencing. This Research Topic was assembled to celebrate that lasting influence. By including four contributions—covering oxidative DNA damage, mitochondrial mutagenesis, nutritional genomics, and advanced sequencing technologies—the Research Topic reflects both the historical development and the future directions of mutation research.

### Oxidative damage and the chemistry of mutation

The review by Cadet and Wagner revisits foundational work on oxidatively generated DNA damage—an area where Ames’ influence was transformative. By recognizing endogenous oxidative stress as a key factor in mutagenesis, Ames helped shift the field beyond a solely exogenous carcinogen framework. DNA damage came to be understood not as an isolated event but as an inherent challenge of aerobic life. This biochemical perspective laid the foundation for the mechanistic analysis of DNA repair pathways and modern lesion-specific analytical techniques. The field’s ability to link specific base lesions with mutational signatures largely depends on this early integration of chemistry and genetics.

### Mitochondrial DNA, aging, and integrated biology

The Opinion article by Dang et al. expands Ames’ intellectual scope into mitochondrial DNA mutagenicity and aging. Mitochondria—centers of metabolism and reactive oxygen species production—serve as a connection between energy generation and genome stability. By integrating oxidative stress, mitochondrial mutation buildup, and aging biology, the authors mirror Ames’ longstanding focus on endogenous damage and nutrient-related defense mechanisms. The work emphasizes a core theme of his career: genomic integrity cannot be separated from metabolic context.

### Nutrition, zinc, and the triage theory

The Mini Review by Wong et al. explores zinc’s protective role in DNA repair and oxidative defense. This review directly aligns with Ames’ triage theory, which suggests that micronutrient deficiencies cause the body to prioritize short-term survival over long-term genome maintenance. Zinc’s function as a structural and catalytic cofactor in DNA repair enzymes offers a molecular basis for this theory. Here, the link between nutrition and mutagenesis becomes clear: dietary status impacts genome stability, which in turn affects aging and disease risk. The framework remains provocative, testable, and increasingly relevant in the era of precision nutrition.

### From the Ames test to duplex sequencing: a technological continuum

The Mini Review by Bhawsinghka and Schaaper—“Detection of mutations: from Ames test to Duplex Sequencing”—traces a progression from bacterial reversion assays to error-corrected next-generation sequencing, with a focus on Duplex Sequencing. It highlights a continuous evolution: (i) The Ames test detects mutations phenotypically via histidine reversion in *Salmonella*; (ii) Reporter assays and transgenic rodent systems expanded detection into mammals; (iii) Next-gen sequencing increased scope but was limited by polymerase errors; (iv) Duplex Sequencing overcame this by tagging and comparing DNA strands, detecting mutations at frequencies of around 10^−8^. The review explains how Duplex Sequencing constructs consensus sequences to distinguish true mutations from artifacts, fulfilling Ames’s original goal: direct, reliable measurement of mutations with mechanistic clarity. While the Ames test offers simplicity and scalability, Duplex Sequencing provides precision and depth. Both share core principles: mutations reflect genotoxic stress; assay sensitivity balances relevance; and mutation spectra, not just frequency, offer mechanistic insights. Importantly, the review also addresses practical limitations—cost, sequencing depth requirements, library-induced artifacts, and challenges in detecting structural variants. These discussions reflect the same pragmatic spirit that made the Ames assay so impactful: methodological rigor coupled with realistic appraisal of constraints.

### Regulatory science and the future of mutagenesis testing

A common thread among these contributions is their regulatory application. The Ames test has become a part of global safety testing standards, influencing Organisation for Economic Co-operation and Development guidelines and pharmaceutical screening protocols. Similarly, Duplex Sequencing is currently being considered for integration into regulatory genotoxicity testing. Widespread adoption of Duplex Sequencing could lead to reduced animal testing, greater sensitivity, and more detailed mutational spectra for risk evaluation. This concept suggests that the field might be experiencing a second major methodological shift—building on Ames’ initial vision and advancing it into the genomic era.

### An expanding framework

Overall, the four contributions in this Research Topic highlight the significant growth of mutagenesis research:

- Covering oxidative base chemistry to mutation landscapes across entire genomes
- From bacterial reversion plates to strand-resolved consensus sequencing
- From nutritional micronutrients to mitochondrial aging
- From regulatory toxicology to clinical mutation detection

The core idea remains clear. Bruce Ames showed us that mutations serve both as a warning sign and a biological story. They cover topics like environmental exposure, metabolic imbalance, repair accuracy, and evolutionary pressure. What has changed is our focus. We now shift from counting revertant colonies to counting individual base substitutions across millions of nucleotides. Yet the core question remains: how do chemical and biological forces influence the genome’s stability?

## Conclusion

This Research Topic does more than honor a pioneering scientist—it shows that his ideas still influence modern research. The Ames test was never just a bacterial assay; it was a statement that mutagenesis could be measured, categorized, and understood mechanistically. From oxidative DNA damage to mitochondrial mutation patterns, from zinc-dependent repair to Duplex Sequencing’s double-strand consensus, the discovery journey is still rooted in that principle. Bruce Ames’ legacy lives on not only in methods but also in mindset: rigorous, integrative, and unafraid to connect molecular detail with human health.

